# Cohort study profile: a cohort of Korean atomic bomb survivors and their offspring

**DOI:** 10.4178/epih.e2024089

**Published:** 2024-11-18

**Authors:** Hamin Lee, Jin-Wu Nam, Mi Kyung Kim, Inah Kim, Yu-Mi Kim, Boyoung Park

**Affiliations:** 1 Department of Preventive Medicine, Hanyang University College of Medicine, Seoul, Korea; 2 Department of Life Science, Hanyang University College of Natural Sciences, Seoul, Korea; 3 Institute of Bioscience and Biotechnology, Hanyang University, Seoul, Korea; 4 Department of Occupational and Environmental Medicine, Hanyang University College of Medicine, Seoul, Korea

**Keywords:** Atomic bomb survivors, Cohort studies, Radiation genetics, Health, Radiation exposure, Nuclear weapons

## Abstract

The Korean Atomic Bomb Survivor Cohort (K-ABC) study was designed to investigate the health impacts of atomic bomb exposure on Korean survivors and to explore whether these effects are passed down genetically to their descendants. This paper outlines the study’s design, data collection methods, baseline socio-demographic characteristics, exposure status, and disease prevalence among the participants, based on survey responses and health examinations. From 2020 to 2024, a total of 2,544 individuals, comprising 1,109 atomic bomb survivors (G1), 1,193 children of G1 (G2), and 242 grandchildren of G1 (G3), consented to participate in the study. Of these, 1,828 participants (659 in G1, 927 in G2, and 242 in G3) completed the survey and underwent health examinations, representing a participation rate of 71.9%. Exposure information was gathered using a questionnaire and verified through records from the Korean Red Cross and a handbook issued by the Japanese government. Disease prevalence was determined based on participants’ self-reported physician diagnoses. This study presents details about the K-ABC study and provides baseline data on the participants recruited. These data will be valuable for interpreting the results of future K-ABC studies.

## INTRODUCTION

Atomic bombs were dropped on the Japanese cities of Hiroshima and Nagasaki in August 1945. These events significantly impacted the many Koreans residing in Japan, who had been sent there as conscripts or laborers in mines and factories, or who had moved there to earn a living. It is estimated that around 70,000 Koreans were affected by the atomic bombings. Of these, approximately 40,000 died due to the bombings, while about 23,000 survivors eventually returned to Korea [1,2].

Since 1950, cohorts of atomic bomb survivors, in utero survivors, and children of survivors have been established in Japan to study the health effects of atomic bomb radiation. The Life Span Study, which includes a cohort of atomic bomb survivors, has documented a significant association between exposure to atomic bomb radiation and a higher incidence of solid cancers [3].

Despite the growing body of evidence on the health effects of radiation exposure in directly exposed individuals, it remains unclear whether these effects are passed down to their offspring. Research involving the offspring of Japanese atomic bomb survivors found no link between parental radiation exposure and increased disease prevalence or *de novo* mutations in their children [4,5]. Conversely, several studies focusing on the Chernobyl disaster have observed elevated genome abnormality rates in the children of Chernobyl liquidators born post-accident, suggesting potential transgenerational transmission of radiation-induced genomic instability [6]. Nonetheless, other research indicates that the impact of transgenerational genetic effects is minimal [7]. As a result, the implications of abnormal genome frequencies on the health of offspring remain unclear.

In Korea, several cross-sectional studies have been conducted on atomic bomb survivors and their children [8–11]. However, the findings from these studies, along with earlier results from research on the offspring of Japanese atomic bomb survivors, have not been sufficient to determine the hereditary effects of radiation exposure. To address this gap, the Korean Atomic Bomb Survivor Cohort (K-ABC) was established to investigate whether the health of survivors and their descendants is linked to exposure to atomic bomb radiation and if there is a hereditary effect from survivors to their offspring. This study introduces the K-ABC by outlining the study design, data collection methods, baseline socio-demographic characteristics, disease prevalence, and overall health status.

## COHORT DESCRIPTION

### Sample size

To estimate an adequate sample size, we set a 2-sided significance level of 95%, an 80% power, an exposed/unexposed ratio of 4, and a prevalence odds ratio ranging from 1.1 to 2.0 in increments of 0.1. Considering the predicted risk size of radiation exposure from the atomic bomb, the magnitude of the risk used to estimate the correlation between general risk factors, and the number of atomic bomb victims that could realistically be investigated, we determined that this study required more than 1,500 participants to accurately reflect the relationship between general risk factors and atomic bomb exposure. Additionally, for trio analysis, it was necessary to recruit 300 participants from the same household. Therefore, to thoroughly investigate the health effects on atomic bomb survivors and the heritability of atomic bomb exposure, at least 1,800 participants were needed for this study.

### Study participants

The K-ABC includes three distinct groups: Korean atomic bomb survivors (G1), who are designated as “victims” under the Special Act on the Support for Korean Atomic Bomb Victims; “G2,” the second generation, which refers to the children of G1; and “G3,” the third generation, including the grandchildren of G1.

The recruitment process for participants in the K-ABC has been previously outlined [12]. Initially, postal mail containing research descriptions and consent forms was dispatched to 2,093 G1 and 2,422 G2 registered members of the Korea Atomic Bomb Casualty Association and the Korea Atomic Bomb Casualty Offspring Association in 2020. Out of these, 1,668 individuals from G1 and G2 (36.9%) returned the signed informed consent forms and were subsequently invited to participate in interviews and health examinations. Additionally, researchers visited health checkup sites designated for G1, which are provided annually by the Korean Red Cross to support the welfare of atomic bomb survivors, to encourage participation in the study. During the baseline survey, individuals from G1 and G2 were encouraged to involve other family members, including parents, siblings, and children, in the study. From 2020 to 2024, a total of 2,544 participants from G1, G2, and G3 were enrolled (1,109 G1, 1,193 G2, and 242 G3). All participants were required to provide consent for their data to be linked to the Statistics Korea mortality data, National Cancer Registry data, National Health Insurance Service data, and Red Cross data via their resident registration numbers. This consent was a mandatory condition for participation and was secured from all 2,544 participants. Of these, 1,828 individuals (659 in G1, 927 in G2, and 242 in G3) participated in both the survey and the health examination, representing a participation rate of 71.9%. If participants met the criteria for a trio as previously defined [12], various configurations of trios were established. Specifically, a complete trio consisted of a G1 participant, their spouse, and at least one of their biological children. Incomplete trios were formed in two scenarios: either a G1 participant or their spouse along with two or more of their biological children participated, or three or more G2 individuals with the same biological parents participated, with at least one being from G1. An internal control trio included a G2 participant, their spouse, and their biological children (G3).

### Variables and measurement

All variables and measurements used in this cohort study are presented in Table 1. The survey items for K-ABC comprised face-to-face interviews, pedigree information, and health examinations. These examinations included anthropometric measurements, blood pressure assessments, blood tests, and urine tests. Whole genome sequencing was conducted on the constructed trios.

#### Questionnaires

The questionnaire encompassed a range of topics including demographic information, exposure to atomic bombs, current health status, diagnosed diseases, history of exposure to medical radiation, health behaviors, and women’s reproductive health. Table 1 presents the items included in the questionnaire. This questionnaire was aligned with other nationwide Korean studies, such as the Korean National Health and Nutrition Examination Survey [13] and the Korean Genome and Epidemiology Study [14]. Unique to the K-ABC, questions related to atomic bomb exposure were specifically directed at G1 individuals who had a history of exposure to medical radiation.

#### Pedigree

The majority of parents of G1 individuals were also exposed to the atomic bomb, and most parents of the surviving G1 and G2 individuals had passed away due to old age. Additionally, those who suffered severe after effects from the atomic bomb tended to die earlier. The G1 participants in this study were generally healthy individuals likely to have a longer lifespan, which could introduce survival bias into the results. To mitigate this bias, detailed pedigree information was collected for both living and deceased family members, including spouses. This information included vital status, cause of death, age at death, history of diagnosed diseases, age at diagnosis, year of birth, and exposure to the atomic bomb. Family members considered included first-degree relatives such as parents, siblings, and children. Furthermore, data on second-degree relatives were gathered, focusing on their vital status, cause of death, and age at death for both G1 and G2. Information about grandchildren was collected solely from G1 individuals, while G2 individuals provided details about their aunts and uncles.

#### Anthropometric measurements and laboratory tests

Height, weight, blood pressure, waist circumference, hip circumference, upper arm circumference, and calf circumference were measured using standardized tools. The assessment included blood and urine tests typically part of a general health examination, such as hemoglobin A1c, random glucose, total cholesterol, triglycerides, low-density lipoprotein, high-density lipoprotein, aspartate aminotransferase, alanine aminotransferase, blood urea nitrogen, creatinine, gamma-glutamyl transferase, cholesterol, urine protein, glucose, and occult blood. Additionally, the K-ABC featured unique tests including white blood cells with differential count, complete blood count, interleukin (IL)-6, IL-4, IL-10, tumor necrosis factor-α, C-reactive protein, erythrocyte sedimentation rate, and interferon-γ, aimed at evaluating immunological and hematopoiesis characteristics specific to K-ABC.

#### Whole-genome sequencing

Targeting participants who met the criteria for trios, next-generation sequencing was used to identify *de novo* mutations and estimate the genetic inheritance of atomic bomb exposure. *De novo* mutations were quantified using the Genome Analysis Toolkit developed by the Broad Institute [14]. Subtypes of *de novo* mutations include single-nucleotide variations, small insertions/deletions, structural variations, copy number alterations, and chromothripsis. Whole-genome sequencing and analysis were conducted on 400 subjects who met the selection criteria established for this study.

### Indirect follow-up of the participants

Through the use of resident registration numbers from participants who provided informed consent, we linked the records of 2,544 participants with data from Statistics Korea mortality, the National Cancer Registry, and the National Health Insurance Service. The vital status and cause of death of individuals from 1992 to 2022 were ascertained using the Statistics Korea Mortality Database, which recorded 81 deaths during the study period. The National Cancer Registry supplied data on cancer prevalence and incidence from 1988 to 2021, identifying 324 individuals with a history of cancer. Medical usage data from 2002 to 2022 were obtained from the National Health Insurance Service, which operates within a single-payer, fee-for-service, and compulsory universal healthcare insurance system.

### Direct follow-up of the participants

Changes in health behaviors, self-reported health, and disease development among participants and their first-degree relatives were assessed using follow-up questionnaires. The initial follow-up is scheduled for 2024 and will be conducted biennially in the future.

### Statistical analysis

Of the 2,544 participants, 1,828 completed the survey; their baseline characteristics, survey responses, pedigrees, and health examinations were analyzed. All categorical variables are presented as frequencies and percentages, while all continuous variables are reported as means and standard deviations. The data were stratified by generation (G1, G2, and G3) for analysis. The chi-square test and Fisher’s exact test were employed to examine disease prevalence based on the survey data from atomic bomb survivors and their descendants. Statistical analyses were conducted using SAS version 9.4 (SAS Institute Inc., Cary, NC, USA).

### Ethics statement

The Institutional Review Board of Hanyang University College of Medicine (approval No. HYUIRB-202007-014-21) approved this study.

## FINDINGS TO DATE

### Baseline characteristics

The 2,544 participants comprised 659 G1, 927 G2, and 242 G3 patients (875 male and 953 female). The baseline characteristics of these 1,828 participants are detailed in Table 2. All individuals in G1 were aged 70 years or older. The most common age range in G2 was 50–59 years, representing 383 (41.3%) of the group. For G3, the predominant age group was 20–29 years, encompassing 90 (37.2%) of its members. A trend was observed where the level of education increased with each successive generation. Additionally, 95.6% of G1 and 84.1% of G2 were affiliated with the Korea Atomic Bomb Casualty Association and the Korea Atomic Bomb Casualty Offspring Association.

Data on the atomic bomb exposure of G1 individuals are shown in Table 3. Among these individuals, 50% could not recall their distance from the hypocenter at the time of the bombing. Consequently, we utilized secondary data from the Korean Red Cross and the *Hibakusha Techo*, a handbook issued by the Japanese government, to determine the variables for exposed place and distance from the hypocenter. Notably, 97.9% of G1 individuals were exposed in Hiroshima. At the time of the bombing, 44.2% of G1 individuals were within 2 km of the hypocenter, classified as “inner proximal” according to the Life Span Cohort of Japanese survivors. Additionally, 18.5% were located 2.1–2.5 km away, termed “outer proximal,” and 32.7% were 2.6–10.0 km away, defined as “distal” by the same cohort. Overall, 84.4% were directly exposed to an atomic bomb, and 8.2% were exposed in utero.

Table 4 presents the health behaviors of the participants, which include smoking and drinking status, subjective health status, exercise frequency, and sleep quality over the past month. Notably, a majority of the participants had never smoked, with 64.5% reporting as such. A comparison revealed that the prevalence of current smokers was higher in the second generation (17.2%) compared to other generations. In terms of drinking status, less than half of the participants were lifetime abstainers (45.0%), with the first generation showing the highest percentage of abstainers at 59.6%. Among the second generation smokers, 55.9% were current smokers. When assessing subjective health status, most participants reported having either moderate (34.2%) or poor (35.0%) health. The first generation reported the highest percentage of poor health at 47.3%. In contrast, the second generation had the highest percentage reporting moderate health (38.7%), and the third generation had the highest percentage reporting good health (38.4%). Regarding exercise frequency, the largest group consisted of individuals who did not engage in physical activity, accounting for 47.5% of responses. The first generation reported the highest frequency of daily exercise (24.0%), while the second and third generations most commonly reported exercising 3–4 times a week (21.7 and 16.9%, respectively). As for overall sleep quality, the response “good” was the most frequently selected by participants, at 49.6%, across all generations.

Table 5 shows the medical radiation exposure history of the participants. Participants reported an average of 17.6 X-ray exposures, making it the most common type of exposure. Interventional radiography had the lowest average exposure, at 3.3 times. The first generation recorded the highest percentage of medical exposure history, followed by the second and third generations. These generations were exposed to various types of medical radiation, including computed tomography, fluoroscopy, nuclear medicine tests, interventional radiography, and radiation therapy. Notably, the second generation had the highest exposure rates to radiography, including dental panoramic radiography and mammography, with average exposures of 7.5 times and 7.6 times, respectively.

Table 6 presents the prevalence of various diseases among participants, categorized by generation. The most common conditions identified in the K-ABC study include hypertension (32.9%), hyperlipidemia (32.2%), vertebral stenosis or disc herniation (30.6%), and cataracts (29.1%). In the G1 group, there was a significant prevalence of several diseases: hypertension, hyperlipidemia, stroke, heart attack/angina, osteoarthritis, rheumatoid arthritis, osteoporosis, vertebral stenosis or disc herniation, diabetes mellitus, benign thyroid disease, cancer, dementia, cataracts, glaucoma, hearing loss, and prostate disease. Conversely, G2 individuals showed a notably high incidence of female infertility, uterine myoma, and vasectomy. In G3, diseases such as atopy and allergic rhinitis were particularly prevalent.

Table 7 shows the exposure data for 26,712 family members of the 1,828 study participants, as collected from the pedigree questionnaire. The data includes information on 10,407 family members from the G1 pedigree, 15,189 from the G2 pedigree, and 1,116 from the G3 pedigree. From this pedigree information, we were able to identify 4,887 individuals from G1, 7,555 from G2, and 5,222 from G3, including both deceased and living members. Regarding exposure to the atomic bomb, 94.8% of G1 fathers and 95.7% of G1 mothers were affected, with over 99% of these individuals subsequently passing away. In G2, 51.7% of fathers and 67.1% of mothers experienced exposure, with mortality rates of 65.4% for fathers and 30.4% for mothers, respectively. Among the siblings in G1 and G2, 57.6% and 8.4%, respectively, were directly exposed to the atomic bomb. The corresponding mortality rates were 41.4% for G1 siblings and 12.9% for G2 siblings.

## STRENGTHS AND WEAKNESSES

This study has the following strengths: First, previous studies of Korean atomic bomb survivors were cross-sectional studies. This is the first cohort study of Korean atomic bomb survivors that collected biospecimens and obtained informed consent for linking different secondary data sources from various organizations, contributing to complete follow-up. Through both direct and indirect follow-ups, long-term health status and disease development can be monitored as the participants age.

Second, this is the first whole-genome study involving a substantial number of atomic bomb survivor trios. Earlier whole-genome studies on atomic bomb survivors and their children, conducted in Japan, included only three trios, an insufficient number to identify any association [5]. Whole-genome sequencing has the potential to reveal direct biological mechanisms underlying the heritability of atomic bomb exposure.

This study has the following limitations: First, several G2 or G3 individuals were unaware that they were offspring of atomic bomb survivors because G1 individuals concealed their exposure from their families due to concerns regarding the stigmatization of their stay in Japan during the colonial era. Additionally, atomic bomb survivors and their descendants who did not possess a *Hibakusha Techo* or were not registered with the Korea Red Cross or Casualty Association were excluded from this study due to the absence of documentation verifying their exposure status.

Second, individuals who died immediately after exposure to the atomic bombs or before the establishment of this cohort study could not be included. This may have led to survival bias. To adjust for this potential bias, the K-ABC obtained pedigree information, including individuals who had already died. Thus, it was possible to examine the effects of atomic-bomb exposure on disease and mortality.

Third, there may have been information bias regarding the exposure of G1 individuals to atomic bombs and their disease history. Since most G1 participants were exposed at a very young age, the accuracy of their recollections may be compromised. To minimize this bias, we linked our data with records from *Hibakusha Techo* and the Korean Red Cross, which provided more precise information about the locations and distances of exposure. However, concerns about information bias for other exposure-related items persist. Similarly, the majority of G1 participants were too young at the time of exposure to remember specific details such as the diseases diagnosed or the dates of diagnosis. To address this limitation, we attempted to link our data with information from the National Cancer Registry and the National Health Insurance Service. However, data from the National Health Insurance Service are only available from 2002, and National Cancer Registry data from 1988 onward. Consequently, any diseases diagnosed before these years could not be captured in our study. Therefore, information regarding the deaths of individuals is required as an alternative indicator of their overall disease status.

This cohort study will continue to monitor the occurrence of death and disease to identify relevant health conditions. Additionally, individuals who did not participate in the study but were included in the pedigree were also tracked to confirm any instances of disease or death.

This study offers insights into the K-ABC study and provides baseline data for the participants recruited. These data could be useful for interpreting results in future K-ABC studies.

## Figures and Tables

**Table 1:** Questionnaires and measurements used in the Korean Atomic Bomb Survivors Cohort

	Generation	No. of items	Sample (n)	Components
Questionnaire	1	273	1,828	Sex, age, association registration status, residential area, family member, marital status, average household income, education; atomic bomb exposure information: hibakusha, place of exposure, distance from the hypocenter, shielding, symptoms after exposure, long-term sequelae; Health status: subjective health, diagnosed diseases; Medical radiation exposure history: X-rays, dental panoramic X-rays, computed tomography, fluoroscopy, mammography, interventional radiography, radiation therapy; Fracture, alcohol consumption, lifetime tobacco smoking, physical activity, sleep duration and quality, nutritional assessment; Reproductive factors: menopausal status, age at menarche, age at first pregnancy, number of delivery experiences, age at first and last deliveries, duration of breastfeeding, age at first breastfeeding, no. of artificial abortions, age at first artificial abortion, hysterectomy history
	2	271		
	3	270		
Pedigree	1	62	1,828	Birth year, vital status, year of death, cause of death, disease history, age at diagnosis, and atomic bomb exposure status of first-degree family (parents, siblings, and children), second-degree family (aunt, uncle, and grandchildren), and spouse; Questions asked to first generation regarding the second-degree family included those on grandchildren; Questions for the second generation regarding second-degree family included those on aunts and uncles; The third generation were not asked any questions regarding second-degree family
	2	62		
	3	45		
Anthropometric	1, 2, 3	11	1,828	Height, weight, waist circumference, hip circumference, upper arm circumference, calf circumference, and blood pressure
Blood examination	1, 2, 3	20	1,784	Random glucose, hemoglobin A1c, aspartate aminotransferase, alanine aminotransferase, gamma-glutamyl transferase, complete blood count, creatinine, blood urea nitrogen, total cholesterol, triglycerides, high-density lipoprotein, and cholesterol
Urine examination	1, 2, 3	10	1,703	pH, nitrate, specific gravity, protein, glucose, ketones, bilirubin, occult blood, urobilinogen, and leukocyte esterase
Whole genome sequencing	Trio (complete, incomplete, and internal trios)			Genome Analysis Toolkit

**Table 2:** Baseline socio-demographic characteristics of participants of the Korean Atomic Bomb Survivors Cohort by generation

Characteristics	Total (n=1,828)	Generation		
		First (n=659)	Second (n=927)	Third (n=242)
Age (yr)				
<20	58 (3.2)	0 (0.0)	0 (0.0)	58 (24.0)
20–29	90 (4.9)	0 (0.0)	0 (0.0)	90 (37.2)
30–39	67 (3.7)	0 (0.0)	6 (0.6)	61 (25.2)
40–49	191 (10.4)	0 (0.0)	164 (17.7)	27 (11.2)
50–59	389 (21.3)	0 (0.0)	383 (41.3)	6 (2.5)
60–69	280 (15.3)	0 (0.0)	280 (30.2)	0 (0.0)
70–79	376 (20.6)	282 (42.8)	94 (10.1)	0 (0.0)
≥80	377 (20.6)	377 (55.2)	0 (0.0)	0 (0.0)
Sex				
Male	875 (47.9)	327 (49.6)	428 (46.2)	120 (49.6)
Female	953 (52.1)	332 (50.4)	499 (53.8)	122 (50.4)
Marital status				
Single	280 (15.3)	0 (0.0)	84 (9.1)	196 (81.0)
Married	1,036 (56.7)	327 (49.6)	673 (72.6)	36 (14.9)
Widowed	319 (17.5)	269 (40.8)	50 (5.4)	0 (0.0)
Separated/Divorced	158 (8.6)	32 (4.9)	117 (12.6)	9 (3.7)
Missing data	35 (1.9)	31 (4.7)	3 (0.3)	1 (0.4)
Education				
Illiterate	127 (6.9)	116 (17.6)	10 (1.1)	1 (0.4)
Primary school^1^	390 (21.3)	259 (39.3)	106 (11.4)	25 (10.3)
Secondary school	200 (10.9)	97 (14.7)	90 (9.7)	13 (5.4)
High school	461 (25.2)	103 (15.6)	317 (34.2)	41 (16.9)
College or more	614 (33.6)	53 (8.0)	400 (43.1)	161 (66.5)
Missing data	36 (2.0)	31 (4.7)	4 (0.4)	1 (0.4)
Registry with the Korea Atomic Bomb Casualty Association				
Registered	1,410 (77.1)	630 (95.6)	780 (84.1)	0 (0.0)
Not registered	176 (9.6)	29 (4.4)	147 (15.9)	242 (100)
Average household income (10^4^ Korean won/mo)				
<100	478 (26.1)	347 (52.7)	121 (13.1)	0 (0.0)
100–299	392 (21.4)	119 (18.1)	235 (25.4)	38 (15.7)
300–499	260 (14.2)	21 (3.2)	195 (21.0)	44 (18.2)
≥500	359 (19.6)	8 (1.2)	283 (30.5)	68 (28.1)
Do not know	339 (18.5)	164 (24.9)	93 (10.0)	82 (33.9)
National basic livelihood security beneficiary				
Yes	95 (5.2)	52 (7.9)	39 (4.2)	4 (1.7)
No	1,703 (93.2)	588 (89.2)	882 (95.1)	233 (96.3)
Do not know	24 (1.3)	17 (2.6)	4 (0.4)	3 (1.2)
Missing data	6 (0.3)	2 (0.3)	2 (0.2)	2 (0.8)
Residential area				
Seoul/Gyeonggi-do/Incheon	480 (26.3)	161 (24.4)	231 (24.9)	88 (36.4)
Busan/Gyeongsangnam-do	962 (52.6)	400 (60.7)	467 (50.4)	95 (39.3)
Deagu/Gyeongsangbuk-do	270 (14.8)	61 (9.3)	167 (18.0)	42 (17.4)
Others	92 (5.0)	20 (3.0)	55 (5.9)	17 (7.0)
Missing data	24 (1.3)	17 (2.6)	7 (0.8)	0 (0.0)

Values are presented as number (%).

1 *Seodang* (a traditional village school in Korea) is categorized as “primary school.”

**Table 3:** Atomic bomb exposure of first-generation individuals in the Korean Atomic Bomb Survivors Cohort (n=659)

Variables	n (%)
Exposed place	
Hiroshima	645 (97.9)
Nagasaki	14 (2.1)
Distance from hypocenter (km)	
0.0–0.5	0 (0.0)
0.6–1.0	14 (2.1)
1.1–1.5	96 (14.6)
1.6–2.0	181 (27.5)
2.1–2.5	122 (18.5)
2.6–3.0	106 (16.1)
3.1–3.5	40 (6.1)
≥3.6	69 (10.5)
Missing data	31 (4.7)
Exposure type	
Direct exposure	556 (84.4)
Intrauterine exposure	54 (8.2)
Entered the city within 2 wk of the bombing	2 (0.3)
Others	1 (0.2)
Missing data	46 (7.0)
Presence of a radiation shield	
None	36 (5.5)
Partial	101 (15.3)
Full	352 (53.4)
Others	2 (0.3)
Missing data	168 (24.5)
Reason for visiting Japan	
Residence	500 (75.9)
Following parents or relatives	77 (11.7)
Others	50 (7.6)
Missing data	32 (4.9)
Symptoms of aftereffects of atomic bomb exposure	
Had aftereffect symptoms	193 (29.3)
Did not have aftereffect symptoms	134 (20.3)
Do not know	302 (45.8)
Missing data	30 (4.6)
Use of medical care due to aftereffects of atomic bomb exposure	
Used	137 (20.8)
Did not use	306 (46.4)
Missing data	216 (32.8)

**Table 4:** Baseline health behaviors, including smoking and drinking, of participants in the Korean Atomic Bomb Survivors Cohort

Variables	Total (n=1,828)	Generation		
		First (n=659)	Second (n=927)	Third (n=242)
Smoking status				
Never smoker	1,179 (64.5)	429 (65.1)	555 (59.9)	195 (80.6)
Former smoker	408 (22.3)	186 (28.2)	213 (23.0)	9 (3.7)
Current smoker	240 (13.1)	44 (6.7)	159 (17.2)	37 (15.3)
Missing	1 (0.1)	0 (0.0)	0 (0.0)	1 (0.4)
Drinking status				
Lifetime abstainer	823 (45.0)	393 (59.6)	331 (35.7)	99 (40.9)
Former drinker	196 (10.7)	108 (16.4)	77 (8.3)	11 (4.6)
Current drinker	805 (44.0)	156 (23.7)	518 (55.9)	131 (54.1)
Missing	4 (0.2)	2 (0.3)	1 (0.1)	1 (0.4)
Health status				
Very good	65 (3.6)	16 (2.4)	20 (2.2)	29 (12.0)
Good	372 (20.4)	82 (12.4)	197 (21.3)	93 (38.4)
Moderate	625 (34.2)	176 (26.7)	359 (38.7)	90 (37.2)
Bad	640 (35.0)	312 (47.3)	299 (32.3)	29 (12.0)
Very bad	125 (6.8)	73 (11.1)	51 (5.5)	1 (0.4)
Missing	1 (0.1)	0 (0.0)	1 (0.1)	0 (0.0)
Exercise frequency (times/wk)				
Never	868 (47.5)	353 (53.6)	386 (41.6)	129 (53.3)
1–2	178 (9.7)	20 (3.0)	123 (13.3)	35 (14.5)
3–4	322 (17.6)	80 (12.1)	201 (21.7)	41 (16.9)
4–6	148 (8.1)	47 (7.1)	83 (9.0)	18 (7.4)
Everyday	311 (17.0)	158 (24.0)	134 (14.5)	19 (7.9)
Missing	1 (0.1)	0 (0.0)	0 (0.0)	0 (0.0)
Sleep quality over the past month				
Very good	204 (11.2)	70 (10.6)	84 (9.1)	50 (20.7)
Good	907 (49.6)	304 (46.1)	477 (51.5)	126 (52.1)
Bad	565 (30.9)	200 (30.4)	303 (32.7)	62 (25.6)
Very bad	116 (6.4)	55 (8.4)	57 (6.2)	4 (1.7)
Missing	36 (2.0)	30 (4.6)	6 (0.7)	0 (0.0)

Values are presented as number (%).

**Table 5:** Baseline medical radiation exposure history of participants and their offspring by generation of the Korean Atomic Bomb Survivors Cohort

Variable	Total (n=1,828)	Generation		
		First (n=659)	Second (n=927)	Third (n=242)
X-rays				
Never been exposed	67 (3.7)	32 (4.9)	15 (1.6)	20 (8.3)
Do not know	559 (30.6)	300 (45.5)	228 (24.6)	31 (12.8)
Missing	0 (0.0)	0 (0.0)	0 (0.0)	0 (0.0)
Exposed	1,202 (65.8)	327 (49.6)	684 (73.8)	191 (78.9)
Mean±SD	17.6±12.9	21.1±13.1	18.0±12.7	10.2±10.0
Dental panoramic X-rays				
Never been exposed	190 (10.4)	95 (14.4)	69 (7.4)	26 (10.7)
Do not know	441 (24.1)	263 (39.9)	152 (16.4)	26 (10.7)
Missing	1 (0.1)	0 (0.0)	1 (0.1)	0 (0.0)
Exposed	1,196 (65.4)	301 (45.7)	705 (76.1)	190 (78.5)
Mean±SD	7.9±6.8	10.7±7.9	7.5±6.4	4.9±4.4
Computed tomography				
Never been exposed	433 (23.7)	71 (10.8)	236 (25.5)	126 (52.1)
Do not know	396 (21.7)	283 (42.9)	99 (10.7)	14 (5.8)
Missing	1 (0.1)	0 (0.0)	1 (0.1)	0 (0.0)
Exposed	998 (54.6)	305 (46.3)	591 (63.8)	102 (42.2)
Mean±SD	6.1±6.7	11.2±7.9	4.2±4.8	2.2±2.0
Fluoroscopy				
Never been exposed	1,098 (60.1)	345 (52.4)	551 (59.4)	202 (83.5)
Do not know	209 (31.7)	65 (7.0)	10 (4.1)	284 (15.5)
Missing	2 (0.3)	1 (0.2)	0 (0.0)	3 (0.2)
Exposed	443 (24.2)	103 (15.6)	310 (33.4)	30 (12.4)
Mean±SD	4.6±6.8	8.3±11.2	3.6±4.4	2.2±2.5
Nuclear medicine test - oral and intravenous administration of radiopharmaceuticals (bone scan, thyroid scan, PET-CT, SPECT, etc.)				
Never been exposed	1,620 (88.6)	536 (81.3)	851 (91.8)	233 (96.3)
Do not know	141 (7.7)	99 (15.0)	34 (3.7)	8 (3.3)
Missing	1 (0.1)	0 (0.0)	1 (0.2)	0 (0.0)
Exposed	66 (3.6)	24 (3.6)	41 (4.4)	1 (0.4)
Mean±SD	5.5±10.3	8.3±16.1	3.8±4.0	8.0±.0.0
Nuclear medicine test - oral and intravenous administration of radiopharmaceuticals (thyroid radioiodine therapy, strontium-89 bone metastasis treatment, etc.)				
Never been exposed	1,677 (91.7)	556 (84.4)	886 (95.6)	235 (97.1)
Do not know	130 (7.1)	94 (14.3)	31 (3.3)	5 (2.1)
Missing	1 (0.1)	0 (0.0)	1 (0.1)	0 (0.0)
Exposed	20 (1.1)	9 (1.4)	9 (1.0)	2 (0.8)
Mean±SD	6.3±17.5	11.8±25.7	1.6±1.3	2.5±2.1
Mammography				
Never been exposed	1,002 (54.8)	342 (51.9)	457 (49.3)	203 (83.9)
Do not know	277 (15.2)	171 (26.0)	101 (10.9)	5 (2.1)
Missing	3 (0.2)	0 (0.0)	3 (0.3)	0 (0.0)
Exposed	546 (29.9)	146 (22.2)	366 (39.5)	34 (14.1)
Mean±SD	8.5±6.4	12.1±7.2	7.6±5.5	3.1±2.6
Interventional radiography				
Never been exposed	1,668 (91.3)	559 (84.8)	872 (94.1)	237 (97.9)
Do not know	102 (5.6)	77 (11.7)	20 (2.2)	5 (2.1)
Missing	1 (0.1)	0 (0.0)	1 (0.1)	0 (0.0)
Exposed	57 (3.1)	23 (3.5)	34 (3.7)	0 (0.0)
Mean±SD	3.3±10.5	5.9±16.3	1.5±1.1	0.0±0.0
Radiation therapy				
Never been exposed	1,686 (92.2)	566 (85.9)	884 (95.4)	236 (97.5)
Do not know	93 (5.1)	72 (10.9)	16 (1.7)	5 (2.1)
Missing	3 (0.2)	1 (0.2)	2 (0.2)	0 (0.0)
Exposed	46 (2.5)	20 (3.0)	25 (2.7)	1 (0.4)
Mean±SD	17.9±22.2	15.8±20.1	20.3±24.1	1.0±0.0

Values are presented as number (%).

SD, standard deviation; PET-CT, position emission tomography-computed tomography; SPECT, single-photon emission computed tomography.

**Table 6:** Disease prevalence among the participants in the Korean Atomic Bomb Survivors Cohort by generation

Variables	Total (n=1,828)	Generation			χ^2^ (p-value)
		First (n=659)	Second (n=927)	Third (n=242)	
Malformations (such as those in the heart and fingers among others)	24 (1.3)	6 (0.9)	12 (1.3)	6 (2.5)	0.186
Cerebral palsy	3 (0.2)	0 (0.0)	3 (0.3)	0 (0.0)	0.379
Hypertension	601 (32.9)	367 (55.7)	230 (24.8)	4 (1.7)	<0.001
Hyperlipidemia	589 (32.2)	274 (41.6)	305 (32.9)	10 (4.1)	<0.001
Stroke	78 (4.3)	53 (8.0)	24 (2.6)	1 (0.4)	<0.001
Heart attack/angina	113 (6.2)	83 (12.6)	30 (3.2)	0 (0.0)	<0.001
Osteoarthritis	308 (16.8)	182 (27.6)	124 (13.4)	2 (0.8)	<0.001
Rheumatoid arthritis	74 (4.0)	35 (5.3)	37 (4.0)	2 (0.8)	0.010
Osteoporosis	287 (15.7)	190 (28.8)	97 (10.5)	0 (0.0)	<0.001
Vertebral stenosis or disc herniation	559 (30.6)	252 (38.2)	285 (30.7)	22 (9.1)	<0.001
Diabetes mellitus	324 (17.7)	195 (29.6)	125 (13.5)	4 (1.7)	<0.001
Benign thyroid disease	212 (11.6)	95 (14.4)	109 (11.8)	8 (3.3)	<0.001
Cancer	198 (10.8)	110 (16.7)	85 (9.2)	3 (1.2)	<0.001
Major depressive disorder	130 (7.1)	55 (8.3)	64 (6.9)	11 (4.5)	0.136
Parkinson’s disease	8 (0.4)	5 (0.8)	3 (0.3)	0 (0.0)	0.309
Epilepsy	4 (0.2)	0 (0.0)	4 (0.4)	0 (0.0)	0.195
Intellectual disability	14 (0.8)	0 (0.0)	10 (1.1)	4 (1.7)	0.003
Peripheral neuropathy	11 (0.6)	6 (0.9)	5 (0.5)	0 (0.0)	0.366
Schizophrenia	4 (0.2)	1 (0.2)	3 (0.3)	0 (0.0)	0.799
Dementia	50 (2.7)	47 (7.1)	3 (0.3)	0 (0.0)	<0.001
Atopy	100 (5.5)	24 (3.6)	42 (4.5)	34 (14.0)	<0.001
Alopecia	85 (4.6)	20 (3.0)	52 (5.6)	13 (5.4)	0.048
Allergy	178 (9.7)	44 (6.7)	111 (12.0)	23 (9.5)	0.002
Allergic rhinitis	273 (14.9)	43 (6.5)	151 (16.3)	79 (32.6)	<0.001
Asthma	87 (4.8)	38 (5.8)	40 (4.3)	9 (3.7)	0.293
Cataract	532 (29.1)	380 (57.7)	151 (16.3)	1 (0.4)	<0.001
Glaucoma	62 (3.4)	39 (5.9)	23 (2.5)	0 (0.0)	<0.001
Macular degeneration	32 (1.8)	18 (2.7)	12 (4.3)	2 (0.8)	0.062
Hearing loss	113 (6.2)	71 (10.8)	40 (4.3)	3 (1.2)	<0.001
Meniere’s disease	14 (0.8)	2 (0.3)	10 (1.1)	2 (0.8)	0.210
Male infertility	4 (0.5)	2 (0.6)	2 (0.5)	0 (0.0)	1.000
Prostate disease	228 (26.6)	166 (53.4)	60 (14.1)	2 (1.7)	<0.001
Vasectomy	96 (11.2)	9 (2.9)	85 (19.9)	2 (1.7)	<0.001
Female infertility	18 (1.9)	1 (0.3)	17 (3.4)	0 (0.0)	<0.001
Uterine myoma	193 (20.6)	44 (13.8)	140 (28.2)	9 (7.4)	<0.001
Tubal ligation	41 (4.4)	16 (5.0)	25 (5.0)	0 (0.0)	0.014
Pain of unknown origin	175 (9.6)	56 (8.5)	103 (11.1)	16 (6.6)	0.053
Other diseases	587 (32.1)	196 (29.7)	338 (36.5)	53 (21.9)	<0.001

Values are presented as number (%).

**Table 7:** Atomic bomb exposure and vital statistics of family members reported by participants in the Korean Atomic Bomb Survivors Cohort, by generation

Variable	Total	Generation		
		First	Second	Third
Subtotal	26,712 (100)	10,407 (100)	15,189 (100)	1,116 (100)
Family member’s generation				
First	4,887 (18.3)	3,505 (33.7)	1,382 (9.1)	0 (0.0)
Second	7,555 (28.3)	3,335 (32.1)	3,973 (26.2)	247 (22.1)
Third	5,222 (19.6)	2,873 (27.6)	1,821 (12.0)	528 (47.3)
Fourth	66 (0.2)	0 (0.0)	0 (0.0)	66 (5.9)
Not exposed	8,904 (33.3)	645 (6.2)	7,984 (52.6)	275 (24.6)
Do not know	78 (0.3)	49 (0.5)	29 (0.2)	0 (0.0)
Sex				
Male	13,255 (49.6)	5,213 (50.1)	7,489 (49.3)	553 (49.6)
Female	13,187 (49.4)	5,106 (49.1)	7,524 (49.5)	557 (49.9)
Do not know	270 (1.0)	88 (0.9)	176 (1.2)	6 (0.5)
Survival status				
Alive	18,921 (70.8)	7,527 (72.3)	10,307 (67.9)	1,087 (97.4)
Dead	7,524 (28.2)	2,804 (26.9)	4,697 (30.9)	23 (2.1)
Miscarriage	233 (0.9)	60 (0.6)	167 (1.1)	6 (0.5)
Stillbirth	8 (0.0)	6 (0.1)	2 (0.0)	0 (0.0)
Do not know	26 (0.1)	10 (0.1)	16 (0.1)	0 (0.0)
Father	1,797 (100)	630 (100)	924 (100)	241 (100)
Exposed history				
Directly exposed	1,075 (59.9)	597 (94.8)	478 (51.7)	0 (0.0)
Indirectly exposed	88 (4.9)	0 (0.0)	0 (0.0)	88 (36.5)
Not exposed	609 (33.9)	21 (3.3)	435 (47.1)	153 (63.5)
Do not know	20 (1.1)	10 (1.6)	10 (1.1)	0 (0.0)
Missing	3 (0.2)	2 (0.3)	1 (0.1)	0 (0.0)
Survival status				
Alive	546 (30.4)	3 (0.5)	320 (34.6)	223 (92.5)
Dead	1,249 (69.6)	627 (99.5)	604 (65.4)	18 (7.5)
Mother	1,795 (100)	630 (100)	924 (100)	241 (100)
Exposure history				
Directly exposed	1,223 (68.1)	603 (95.7)	620 (67.1)	0 (0.0)
Indirectly exposed	160 (8.9)	0 (0.0)	1 (0.1)	159 (66.0)
Not exposed	396 (22.1)	14 (2.2)	300 (32.5)	82 (34.0)
Do not know	14 (0.8)	11 (1.8)	3 (0.3)	0 (0.0)
Missing	2 (0.1)	2 (0.3)	0 (0.0)	0 (0.0)
Survival status				
Alive	885 (49.3)	4 (0.6)	643 (69.6)	238 (98.8)
Dead	910 (50.7)	626 (99.4)	281 (30.4)	3 (1.2)
Brothers and sisters	6,463 (100)	2851 (100)	3326 (100)	286 (100)
Exposure history				
Directly exposed	1,921 (29.7)	1,643 (57.6)	278 (8.4)	0 (0.0)
Indirectly exposed	4,438 (68.7)	1,133 (39.7)	3,020 (90.8)	286 (100)
Not exposed	72 (1.1)	59 (2.1)	13 (0.4)	0 (0.0)
Do not know	31 (0.5)	16 (0.6)	15 (0.5)	0 (0.0)
Survival status				
Alive	4,838 (74.9)	1,661 (58.3)	2,893 (87.0)	284 (99.3)
Dead	1,612 (24.9)	1,181 (41.4)	429 (12.9)	2 (0.7)
Do not know	13 (0.2)	9 (0.3)	4 (0.1)	0 (0.0)
Children	4,087 (100)	2,188 (100)	1,833 (100)	66 (100)
Exposure history				
Directly exposed	1 (0.0)	1 (0.0)	0 (0.0)	0 (0.0)
Indirectly exposed	4,085 (99.9)	2,186 (99.9)	1,833 (100)	66 (100)
Not exposed	1 (0.0)	1 (0.0)	0 (0.0)	0 (0.0)
Survival status				
Alive	3,695 (90.4)	2,003 (91.5)	1,632 (89.0)	60 (90.9)
Dead	151 (3.7)	119 (5.4)	32 (1.8)	0 (0.0)
Miscarriage	233 (5.7)	60 (2.7)	167 (9.1)	6 (9.1)
Stillbirth	8 (0.2)	6 (0.3)	2 (0.1)	0 (0.0)

Values are presented as number (%).
